# Electrophysiological signatures of memory reactivation in humans

**DOI:** 10.1098/rstb.2019.0293

**Published:** 2020-04-06

**Authors:** Thomas Schreiner, Tobias Staudigl

**Affiliations:** 1 School of Psychology and Centre for Human Brain Health, University of Birmingham, Birmingham, UK; 2 Department of Psychology, Ludwig-Maximilians-University Munich, Munich, Germany

**Keywords:** replay, memory reinstatement, consolidation, retrieval, electroencephalography/magnetoencephalography/intracranial electroencephalography, targeted memory reactivation

## Abstract

The reactivation of neural activity that was present during the encoding of an event is assumed to be essential for human episodic memory retrieval and the consolidation of memories during sleep. Pioneering animal work has already established a crucial role of memory reactivation to prepare and guide behaviour. Research in humans is now delineating the neural processes involved in memory reactivation during both wakefulness and sleep as well as their functional significance. Focusing on the electrophysiological signatures of memory reactivation in humans during both memory retrieval and sleep-related consolidation, this review provides an overview of the state of the art in the field. We outline recent advances, methodological developments and open questions and specifically highlight commonalities and differences in the neuronal signatures of memory reactivation during the states of wakefulness and sleep.

This article is part of the Theo Murphy meeting issue ‘Memory reactivation: replaying events past, present and future’.

## 1. Introduction

Episodic memory, the capacity to remember and relive past experiences, represents a cornerstone of human cognition and the sense of our self [1]. Our memories allow us to reflect about the past and to guide our behaviour for future challenges. The neural mechanisms promoting these abilities, thus the encoding, storage and retrieval of memories, have attracted considerable scientific interest during the last decades.

It is assumed that the hippocampus and the neocortex collaborate tightly to form episodic memories [2–4]. New experiences are encoded by distributed neocortical learning networks and are associated with hippocampal sequences. These sequences organize the networks' input in a way that they can be thought of as sparse and distinct representations of an event's trajectory [5]. They are formed in a very rapid, one-shot type of learning manner enabled by recurrent networks in the CA3 subfield [6].

During retrieval, the hippocampal sequences associated with a given experience are thought to be triggered upon arrival of a retrieval cue (i.e. a part of the sequence), pointing towards the neocortical assemblies that represented the events during their initial perception [5,7]. The successful reinstatement of these representations completes the process of memory reactivation (see box 1) and is assumed to be necessary for the conscious access during episodic remembering [9].

Box 1. **Glossary.**

The terms replay, reactivation and reinstatement are not consistently used across studies of memory reactivation. The following is a suggestion to clarify their use in the current review.

Memory reactivation: A process that leads to the activation of a memory trace or engram. Comprises reinstatement or replay and pattern completion.

Reinstatement: Observation that activity patterns that were present during a past experience reoccur. Often used in conjunction with a cortical site (cortical reinstatement).

Replay: Observation that experience-induced sequences of neuronal activity reoccur. Often implies a statement about temporal dynamics and order: forward versus backward replay; non-compressed or time-compressed replay. The term is most commonly associated with rodent studies that investigate the replay of hippocampal firing patterns in relation to an animal's spatial trajectories.

Pattern completion: A process ascribed to the autoassociate networks of the hippocampal CA3 region. It has been proposed to be crucial for successful episodic remembering because it enables the activation of a previously established association (e.g. place-object) by a partial retrieval cue (e.g. place) [6,8].

Retrieval: A process initiated by an internal or external cue, that can result in memory reactivation and, if successful, in episodic remembering. Includes other processes such as selection and evaluation (not discussed here, but see e.g. [1]).

Episodic remembering/recollection: Observation that past events are remembered with detailed contextual information about a personal experience (e.g. via verbal report during recall).

Memory Consolidation: Observation that learning experiences are strengthened during post-encoding ‘offline’ periods.

During subsequent sleep, hippocampal sequences spontaneously trigger the memory reactivation in the neocortex. The repeated reactivation of memory traces during sleep is assumed to transform them from initially labile to long-lasting representations, permanently stored in neocortical networks [10] (for an alternative hypothesis concerning the role of sleep in memory consolidation please see [11]).

Spearheaded by rodent models, it has become increasingly clear that such hippocampal-triggered memory reactivation might indeed constitute a common mechanism enabling both the retrieval of memories during wakefulness and the strengthening (i.e. consolidation) of memories during sleep [12]. Initial evidence originated from research on memory consolidation, investigating how the brain strengthens memories during offline periods such as sleep. These pioneering studies revealed that the sequences of neuronal firing which occurred during exploration of a novel environment in the hippocampus, are spontaneously reactivated during subsequent non-rapid eye movement (NREM) sleep [13,14]. The magnitude of the reactivation predicted future memory performance [15]. Intriguingly, reactivation activity during sleep has been found beyond the hippocampus in various other brain regions (e.g. prefrontal and visual cortex, ventral striatum [16–18]), with a tight coordination between hippocampal and neocortical reactivation signals [19,20]. Successive work indicated that memory reactivation is not limited to consolidation processes acting during sleep but also emerges while the animals are awake (i.e. during immobility, consummatory behaviour, grooming and task engagement [21]). As sensory input has been shown to trigger and bias hippocampal and accompanying neocortical reactivation processes, it has been suggested that they might also subserve memory retrieval, thereby enabling memory-guided decision making [12].

While these findings without doubt represent important breakthroughs, a pressing question is whether comparable processes guide the multi-facetted expressions of human memory. Beyond that, investigating reactivation processes and their significance for memory in humans represents an invaluable perspective by itself. It not only allows for assessing the cognitive processes associated with reactivation processes in a fine-grained manner but ultimately meets the requirements for assessing the multi-sensory aspects of real-life experiences and memories.

In this review, we summarize and highlight recent advances to capture and characterize memory reactivation in human electrophysiology. We will specifically, but not exclusively, address the role of human memory reactivation in the context of memory retrieval during wakefulness and consolidation acting during sleep, and discuss their potential commonalities, while defining open questions and future directions.

## 2. Memory reactivation in awake humans

The hallmark of human episodic memory is the capacity to remember past events with rich contextual details and conscious access, such that the subjective experience of remembering can be described as mentally travelling to the past. The reactivation of a memory trace of a past event is assumed to be necessary to achieve this subjective experience of remembering. Tulving [9] describes the act of retrieval as a correlation of the information provided by a reminder (memory cue) with the initially experienced information stored in the memory trace. Accordingly, an effective memory cue should overlap with encoded events to trigger a correlation between the activity present during perception and the retrieval attempt. Behaviourally, this has been demonstrated using context-dependent memory paradigms: the probability of correctly remembering an item is higher when study and test contexts match, when compared with when they do not match (e.g. [22–24]). These effects have been integrated in cognitive theories such as the Encoding Specificity Principle ([25], see also [26]) or transfer appropriate processing [27].

### (a) How memory reactivation can be studied in the human brain

As outlined above, memory reactivation can be operationalized as a correlation between activity during the initial perception of an event (for example, a study phase) and the activity patterns recorded during the retrieval of this event (usually a test phase). Although spontaneous retrieval of past events occurs during rest, most human studies investigated memory reactivation during intentional retrieval attempts, providing either complete (recognition) or partial (cued recall) reminders of a specific event or no specific reminders at all (free recall). If reactivation drives memory retrieval, the neural activity associated with a reminder/cue that successfully triggers retrieval of past events (a memory) should resonate with the neural footprint of these events, which was established during encoding (please see box 2 exemplifying a procedure to isolate reactivation processes from neural signals). Importantly, multivariate analysis methods have been shown to be capable of detecting such similarities in multidimensional brain recordings. Among the most commonly used metrics are classification accuracies (e.g. training a classifier on study activity to relate brain activation patterns with experimental conditions and test its parameters on the test data) and pairwise measures of similarity (e.g. Pearson's correlation, cosine similarity), which provide the input to a representational similarity analysis (RSA; [30]). While pioneering work on memory reactivation has been conducted using functional magnetic resonance imaging (fMRI) (e.g. [31]), this review will focus on electrophysiological research, comprising electroencephalography (EEG), magnetoencephalography (MEG), as well as intracranial EEG (iEEG; including depth electrodes and electrocorticography) and single-unit recordings. Such electrophysiological recordings provide exceptionally rich data, which allow for assessing neural similarities in time or space, for fixed frequencies or dynamic time-frequency patterns, for oscillatory power and phase, and the firing behaviour of single units and populations of units.

Box 2. **Exemplary procedure to isolate memory reactivation using similarity measures.**

To identify memory reactivation processes in humans, neural activity is compared between different states or processes (e.g. encoding versus retrieval, retrieval versus targeted memory reactivation). In this example based on Schreiner *et al.* [28], item-specific reactivation processes during sleep elicited by targeted memory reactivation were investigated. Electroencephalography (EEG) was performed while participants retrieved word pairs and subsequently slept for 3 h. During NREM sleep, some of the prior learned words cues were repeatedly presented to experimentally induce memory reactivation. To detect content-specific memory reactivation, the similarity in oscillatory phase [29] between wakefulness and sleep was compared. The assumption here is that the observed similarity between states indicates the reactivation of memory traces. As apparent in the phase similarity plot (bottom right), memory retrieval-related phase patterns re-emerged during targeted memory reactivation (TMR) in a re-current fashion, indicating the reactivation of memories during sleep. Importantly, for such a rationale to work, spurious correlations that are driven by the perceptual identity of the used stimuli need to be excluded. The example illustrates how this objective might be achieved (please note that this procedure just represents one possibility to preclude spurious effects). The elements on the diagonal of the confusion matrix depict similarities between identical stimulus presentations during retrieval and targeted memory reactivation. By comparing subsets of these elements (e.g. remembered versus forgotten), differences driven by perceptual identity should be eliminated. Hence, if remembered items show a higher similarity between retrieval and targeted memory reactivation than forgotten items, as depicted in the phase similarity plot, the effect cannot be attributed to differences of the presented stimuli.

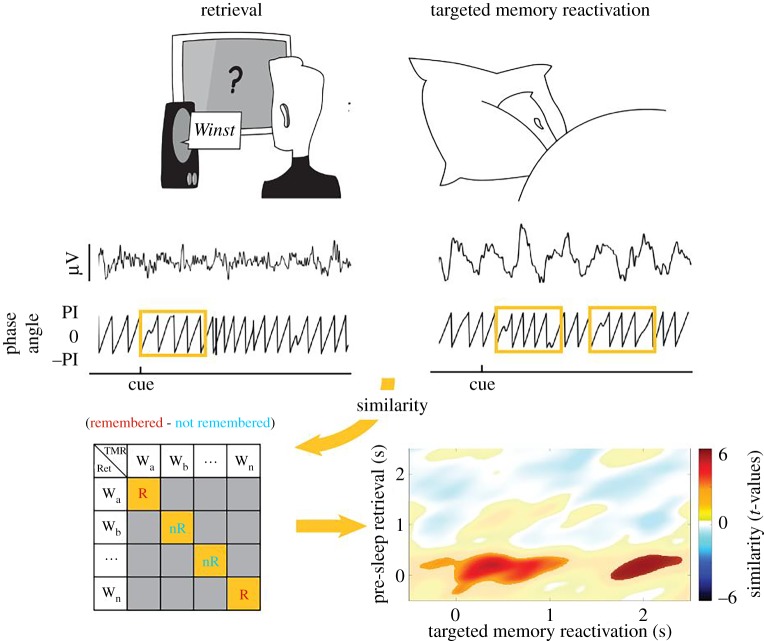

### (b) Content-specific memory reactivation during retrieval

#### (i) Oscillatory signatures of memory reactivation

Recording techniques on a meso and macro level (MEG, EEG, iEEG) have widely adopted an oscillatory perspective on the investigation of memory reactivation. The idea that brain oscillations provide the means to track memory reactivation is grounded in the notion that oscillations organize cell assemblies to encode information [32] and coordinate neural communication and plasticity [33]. Assuming that the frequency and phase of oscillations represent information and cognitive operations at a high level of specificity [34–38], one should be able to identify the reactivation of such content-specific oscillatory patterns during memory retrieval. Because the investigation of similarities in oscillatory activity between encoding and retrieval offers ways to study the neural underpinnings of memory reactivation on multiple levels with several methods, oscillations became a major target in searching for the neural mechanisms underlying memory reactivation.

*Non-invasive studies.* Over the past 5 years, a growing number of studies exploited the possibilities of non-invasive EEG/MEG recordings to investigate memory reactivation during memory retrieval. Among the first to do so, Jafarpour *et al*. [39] applied multivariate pattern classifiers to spatial patterns in time-frequency resolved MEG data to discriminate different categories (faces versus scenes) during encoding. The authors then tested the classifiers' performance on the retrieval data. This procedure enabled them to identify an early reactivation of category-specific encoding activity during cued recall. A similar approach was applied by Johnson *et al*. [40], who trained a multivariate pattern classifier on the time-series of EEG data to distinguish three different encoding tasks involving word learning, and tested its performance on recognition data. Increased classifier performance for correctly recognized versus missed items was taken as an indicator that encoding patterns were indeed reactivated. In general, pattern classifiers offer a versatile tool to study memory reactivation (see [41,42] later in this review, for recent applications), but are limited when it comes to tracking multiple item-specific memory representations. A different approach was taken by Wimber *et al*. [43] who entrained different frequencies via flickering words at encoding, with the aim to recover those frequencies during a subsequent memory test. They found that the imposed frequencies re-emerged when participants successfully recognized a previously learned word (but see [44,45] for conflicting findings).

While these studies identified category-specific memory reactivation, employing RSA on EEG/MEG data enabled the tracking of item-specific memory reactivation. Staudigl *et al*. [46] investigated the effects of a context match/mismatch on the reactivation of item-specific MEG signals. They found a reactivation of video-scene context in cortical areas indexed by beta band (30 Hz) activity, using temporal and spatial pattern similarity analyses. As predicted by the Encoding Specificity Principle [25], the neuronal pattern of context reactivation was reversed in the context mismatch condition: higher reactivation for later forgotten then later remembered items. Building on these findings, Staudigl & Hanslmayr [47] tested whether similar reactivation patterns can be identified in a sensory match/mismatch memory paradigm. The authors found that neural pattern reinstatement of MEG activity at 6–8 Hz benefitted memory only in the match condition, but impaired memory in the mismatch condition. However, a reversal of memory reactivation in the match versus mismatch condition was only obtained for aurally but not visually encoded words. Staudigl and Hanslmayr employed a phase-based similarity analysis introduced by Michelmann *et al*. [29]. In their study, Michelmann and colleagues could show that the phase of low-frequency activity at approximately 8 Hz indexed the item-specific reactivation of memory traces. Importantly, EEG source reconstruction indicated that modality-specific regions (auditory and visual cortex) displayed the most pronounced reactivation. Item-specific reactivation patterns in low-frequency phases were also found in EEG scalp recordings by Schreiner *et al*. [28] comparing two consecutive instances of memory retrieval. To track the speed and time scale of memory reactivation, Michelmann *et al*. [48] recorded MEG while participants remembered movie scenes consisting of multiple sub-events. The authors reported evidence for a flexible switching between compressed and non-compressed forward reactivation of sequences during memory reactivation. Consistent with their previous studies [29,49], memory reactivation was captured by the phase of oscillatory activity at 8 Hz.

The aforementioned correlational findings received further support from studies probing the importance of memory reactivation for retrieval with transcranial alternating current stimulation (tACS) and repetitive transcranial magnetic stimulation (rTMS). Waldhauser *et al.* [50] applied rTMS to posterior brain areas where memory reactivation during retrieval was identified via a pattern of lateralized alpha/beta desynchronization. rTMS interfered with the source memory accuracy during retrieval, suggesting that intact reactivation might be causal for successful memory retrieval. Using tACS to entrain rhythmicity during the encoding and retrieval of a memory task, Javadi *et al*. [51] could show that memory performance was enhanced when tACS frequencies during encoding and retrieval were matched, providing further evidence for the existence of the encoding specificity principle on a neural level.

Taken together, there is now converging evidence from non-invasive EEG/MEG studies for item-specific memory reactivation during retrieval. Specifically, the phase of low-frequency oscillatory activity has shown to be particularly capable of tracking content-specific memory reactivation [28,29,47,48].

*Invasive studies.* Intracranial recordings have some clear advantages over non-invasive techniques that record activity outside the brain: a higher spatial resolution allows more reliable inference of the source of the activity, in particular, for deep brain structures as the hippocampus. Intracranial recordings usually have a higher signal-to-noise ratio, which benefits the investigation of low-amplitude signals (e.g. gamma-band activity above 40 Hz). On the downside, intracranial data usually comes with a lack of a whole-brain perspective and is limited to patient populations in clinical settings. Pre-surgical epilepsy patients, for example, may suffer from a serious neurological disorder, such as hippocampal sclerosis, and generally require a strict medication regime. Both factors presumably impact the outcomes of these studies. Nevertheless, iEEG studies represent a valuable approach to study reactivation processes in the human brain, especially if their outcomes may be aligned with rodent models and non-invasive neuroimaging techniques.

Exploiting the high signal to noise in their data, many iEEG studies have focused on activity in the gamma band. Zhang *et al*. [52] investigated the reactivation of gamma power (42–120 Hz) recorded during the encoding and retrieval of a virtual-navigation paradigm. Using RSA, they found that encoding-retrieval correlations globally increased for remembered spatial paths. In a similar vein, Yaffe *et al*. [53] reported reactivation of distributed cortical gamma power (62–100 Hz) during cued recall of previously learned paired associates.

Exploiting the high spatial specificity of intracranial recordings, Staresina *et al*. [54] used RSA to investigate the pattern similarity between the encoding and retrieval of word-colour associations in the human hippocampus. Successful retrieval of the association was accompanied by an increase in representational similarity when compared with when the association was not retrieved (albeit successful item recognition). The authors interpreted this result as reflecting hippocampal pattern completion processes. Estefan *et al*. [55] recorded from the hippocampus and the lateral temporal cortex and could show that item-specific reinstatement in the lateral temporal cortex was preceded by the reactivation of item-context associations in the hippocampus, which could be described as pattern completion. They also show increased levels of cortico-hippocampal communication alongside the reinstatement periods, which is in line with the idea that the hippocampus triggers cortical reinstatement during memory reactivation. Combining hippocampal depth electrodes and electrocorticography from five patients, Lohnas *et al*. [56] found that high-frequency activity (45–115 Hz) indexed memory reactivation in the hippocampus and the occipitotemporal cortex. Since they manipulated the similarity of encoded items, they could show evidence for pattern separation (i.e. a process facilitating the distinct representation of highly related events [8]): the hippocampal response differentiated new items that were similar to old items. This finding is of particular interest since the hippocampus has been suggested to perform both pattern completion and pattern separation [8].

Intracranial recordings also provide the opportunity to investigate electrophysiological signatures that are challenging to track with non-invasive methods, such as high-frequency ripples. Rodent studies indicated that high-frequency ripples play a crucial role for the temporal coordination of memory reactivation [57–60]. A recent study by Vaz *et al*. [61] provided first evidence for a role of high-frequency ripples for memory reactivation in awake humans. They identified high-frequency ripples (80–120 Hz) in intracranial recordings and showed that the coupling of ripples in the medial temporal lobe and the middle temporal gyrus was increased during successful memory retrieval. Immediately after the coupled ripples, increased similarity between encoding and retrieval broadband activity in the middle temporal gyrus indicated memory reactivation during successful retrieval. Another recent iEEG study supports the importance of hippocampal ripples and memory reactivation for successful memory retrieval. Using a free recall paradigm, the authors show that the number of hippocampal high-frequency ripples (70–180 Hz) increased during the successful encoding of visually presented items as well as prior to their recollection [62]. Importantly, recollection was accompanied by content-specific memory reactivation in visual areas, which in turn was coupled to ripple emergence. These findings provide an important link of human awake memory reactivation to studies in rodents, while emphasizing the functional role of hippocampal high-frequency ripples in memory processes [57,58,60,63–65].

In sum, invasive studies provide convincing evidence for the content-specific reactivation of oscillatory activity during memory retrieval. Importantly, their spatial specificity in conjunction with their high temporal resolution provide the basis for understanding the cascade of processes involved in memory reactivation. Converging iEEG evidence now supports animal and computational models describing the interactions between hippocampus and cortex during memory reactivation.

#### (ii) Single neuron activity reveals timing and circuitry of memory reactivation

Compared to the meso- and macroscopic recordings of brain activity described above, recording single-unit activity in human epilepsy patients provides the opportunity to investigate the neural operations of memory reactivation in neural microcircuits. Multiple studies have now shown that single-unit firing patterns recorded during the presentation of stimuli re-occur during their successful retrieval: Gelhard-Sagiv *et al*. [66] reported that the selective firing of hippocampal and entorhinal single units during the presentation of audiovisual scenes re-occurred during their successful recall. Jang *et al*. [67] showed that unique spiking activity patterns representing words in the anterior temporal lobe were reinstated when successfully retrieving those words from memory. Inspired by animal work on navigation, Miller *et al*. [68] recorded single-unit activity while patients performed a virtual reality navigation task that was paired with a memory test. The authors identified single units in the medial temporal lobe firing at specific virtual locations. Strikingly, when patients remembered items associated with these locations, the place-responsive firing of the single units was reactivated.

In a recent study, Staresina *et al*. [69] recorded single-unit activity from the human entorhinal cortex and the hippocampus in patients performing an associative memory task. Successfully remembered associations could be decoded from population codes in the entorhinal cortex, but not the hippocampus. Hippocampal firing rates, however, predicted the strength of the entorhinal reactivation. The authors interpreted this cascade of neural activity in the medial temporal lobe as reflecting the basic ingredients of a pattern completion process: the hippocampus coordinates reactivation of the encoded information in the (entorhinal) cortex.

### (c) Reactivation of temporal context—neural contiguity

While the studies described above focused on the reactivation of content-specific information during memory retrieval, a different approach to memory reactivation is investigating neural correlates of shifting temporal context. Studies following this approach exploit the fact that contextual representations gradually change over time, that is, that events that are in temporal proximity should share a more similar context than events that are far apart in time. Behaviourally, this has been described as the contiguity effect during free recall, where items that were presented in temporal proximity during encoding are also recalled in temporal proximity [70,71]. In neural terms, this means that remembering an item would elicit brain activity associated with other items presented in close temporal proximity. Such neural contiguity has been shown by Manning *et al*. [72] in electrocorticography recordings. The degree to which distributed patterns of oscillatory activity was similar between items decreased with increasing positional distance of their initial presentation during encoding. Similarly, Yaffe *et al*. [73] found that successful retrieval in a paired-associate memory paradigm involves the reactivation of a gradually changing neural signature during encoding. Manning *et al*. [74] found that neural signatures of semantic reactivation were related to semantic clustering of items during recall, suggesting that a neural contiguity effect is not restricted to the temporal domain. Further support for the reactivation of contextual features during successful memory retrieval comes from studies recording single units in humans. Based on population vector analyses, Howard *et al*. [75] and Folkerts *et al*. [76] showed that the temporal context during encoding was reactivated during successful recognition. These findings support the idea that the reactivation of fluctuating temporal contexts could be used to temporally differentiate past episodes and allocate oneself in time while mentally travelling to the past [71].

### (d) Reactivation in studies not involving episodic memory retrieval

While the present review focuses on memory reactivation during retrieval, electrophysiological signatures of memory reactivation have also been demonstrated during encoding, working memory maintenance, goal-directed navigation and rest. Comparing consecutive instances of encoding, increased similarity for repeated presentations of the same item was found to be predictive of subsequent memory performance [77,78]. The assumption in these studies is that a repeated presentation of an item implicitly triggers retrieval of the memory of the initial presentation. Implicit memory reactivation can also be identified at event boundaries: during sequential learning, prior events were reactivated when the context changed, presumably to link adjacent representations with the goal to form a coherent, continuous episode in long-term memory [79].

Neuronal similarity was also found between activity during encoding and maintenance in working memory [49,80–82]. Here, rather than retrieving representations from memory, they are assumed to be held active for subsequent output. Rose *et al*. [83] reported that even unattended items can be briefly reactivated in working memory by single-pulse TMS.

A recent study investigated memory reactivation during goal-directed navigation [84]. Analogous to working memory, representations had to be maintained by the participants while they navigated towards a goal in a virtual reality environment. Recording iEEG in epilepsy patients, the authors could show that the neuronal representations of cue-goal associations were reactivated during specific phases of hippocampal and frontal theta activity. This finding is in line with the view that a function of the hippocampal theta phase is to temporally segregate information [5,85].

Liu *et al*. [86] recently demonstrated memory reactivation during rest. Analysing MEG data, the authors found time-compressed replay of previously learned abstract sequences, in line with a previous MEG study [87]. The replay was accompanied by ripple-like activity localized to the medial temporal lobe and the direction of the replay was reversed after a sequence was associated with reward. These findings from non-invasive human MEG activity very accurately resemble replay patterns found in rodents [88].

## 3. Memory reactivation during sleep

Over the last decades, it has become increasingly clear that mnemonic processes continue beyond ‘online’ encoding and retrieval processes. Specifically, behavioural research has shown that post-learning sleep is inevitable for the stabilization of newly formed memories and critically supports memory formation [10]. The strengthening of learning experiences during post-encoding ‘offline’ periods, called consolidation [89], has been consistently demonstrated for diverse types of memories including priming, conditioning and procedural motor memories [90–92], as well as episodic memories [93,94]. Theoretical accounts have long postulated memory reactivation as a mechanism mediating the beneficial effects of sleep on memory performance. However, as reactivation processes in sleeping humans are notoriously hard to capture, empirical evidence directly supporting this notion remained scarce until recently. The forthcoming sections will briefly summarize the memory function of sleep and its neural underpinnings. We will specifically address the role of memory reactivation in this context and highlight recent advances and approaches to identify reactivation processes during human sleep.

### (a) The memory function of sleep

What is the advantage to exploit sleep for memory consolidation? Stable memory traces originating from specific events and experiences are assumed to be encoded as long-lasting physical change within distributed learning-related neocortical circuits [95]. A major challenge to this proposal is how these physical changes can emerge during wakefulness, while the brain's capabilities are required to process a continuous stream of new experiences. The standard two-stage model of memory offers an elegant solution to this dilemma [2,9]. It puts forward that new experiences are encoded in parallel in the hippocampus and neocortical learning networks. During subsequent sleep, a period without the necessity to process external events, the hippocampus spontaneously reactivates the prior-encoded memories, thereby coordinating memory reactivation in the neocortex. The repeated reactivation of memory traces is assumed to transform them from initially labile to long-lasting representations, permanently stored in neocortical networks.

In contrast to wakefulness-related retrieval processes, an intricate interplay of sleep-specific oscillations seems to mediate the ‘hippocampal-neocortical dialogue’ during sleep. Put forward by the ‘active systems consolidation theory’, a precise temporal coupling of hippocampal ripples and the two cardinal oscillations of NREM sleep, namely slow oscillations (SOs) and sleep spindles, constitutes the mechanistic vehicle of sleep-related memory consolidation [96]. In this context, the SOs (<1 Hz), which are generated in neocortical circuits [97], have been ascribed the role of a time-giving pacemaker: their depolarizing up-states are assumed to drive the reactivation of memories in the hippocampus, in parallel with hippocampal ripples and thalamo-cortical sleep spindles (waxing and waning oscillatory events between 11 and 15 Hz) [98]. Through this synchronization, SOs provide a crucial basis for the information exchange between the hippocampus and learning-related neocortical areas [10]. Importantly, the coupling of SOs and spindles has also been shown to establish an environment facilitating synaptic plasticity and by that allow long-lasting memory representations to establish in cortical circuits [99,100]. Thereby sleep-related oscillations might counteract the generally unfavourable circumstances for synaptic plasticity present during NREM sleep, which is due to attenuated neuromodulatory activity [101].

Bolstering these assumptions, a close relationship of the cardinal sleep oscillations (ripples, spindles and SOs) with consolidation processes has already been established, both in animal models and humans [102]. It has been shown that intense learning increases the ensuing incidence of sleep spindles, SOs and ripples during NREM sleep [103–105], while their occurrence in return reliably predicts the degree of consolidation [106–108].

Despite these correlational results, more direct evidence embracing the importance of sleep-related oscillations in service of consolidation stems from studies directly manipulating their characteristics. Selectively enhancing SO activity during human sleep via auditory closed-loop stimulation has been demonstrated to simultaneously boost SO-coupled spindle activity and declarative memory performance [109]. Similarly, in rodent models, amplifying the endogenous interplay of hippocampal ripples, SOs and sleep spindles by electrical stimulation resulted in the fortified consolidation of labile memory traces [110]. By contrast, suppressing hippocampal ripples actively disturbed spatial memory formation [111]. Still, according to theoretical accounts, the interplay of hippocampal ripples, thalamo-cortical spindles and cortical SOs might just represent the mechanistic vehicle mediating memory consolidation during sleep [10]. Ultimately, the actual mnemonic representations may need to be reactivated and strengthened, giving rise to long-living memory engrams.

### (b) Memory reactivation during sleep in humans

Compared to rodent models, access to the content of memory re-processing during sleep in humans is more limited due to methodological constraints, rendering the assessment of memory traces a challenging endeavour. Early efforts to reveal memory reactivation during sleep in humans hence mainly examined whether brain regions involved in the initial experience would show corresponding activity during ensuing sleep, using fMRI and positron emission tomography. Indeed, brain activation associated with the learning of diverse memory contents (declarative and motor learning, spatial navigation) was shown to re-appear during ensuing sleep [112–114], with the putative reactivation-signatures peaking during the presence of sleep spindles [114]. Furthermore, the magnitude of these reactivation events predicted the behavioural consolidation benefits, suggesting their functional significance. Hence, in accordance with rodent models, these studies provided first evidence that reactivation-like processes might take place during human NREM sleep, potentially serving its memory function [115]. Still, the used brain imaging techniques chronically suffer from their rather low temporal resolution, fundamentally impeding the fine-grained characterization of memory reactivation and its conveying mechanisms (but see [116]). In addition, although representing important breakthroughs, these early studies did not directly assess the similarity/overlap between the learning-related brain activation and potential reactivation of distinct memory content, which constitutes a core assumption of memory reactivation.

#### (i) Identifying memory reactivation using multivariate approaches

As outlined above, research on the formation of memory engrams and the putative role of memory reactivation during sleep was traditionally spearheaded by invasive animal electrophysiology. However, the advent of multivariate analyses methods, in particular, multivariate pattern analysis (MVPA [117]) and representational similarity analysis (RSA [118]) constituted a fundamental game-changer in human research. As outlined above, these methods allow for estimating item-specific brain patterns, which represents an essential prerequisite for identifying unique memory traces and their reactivation. The following section will delineate pioneering attempts to capture and track reactivation processes during sleep in humans by applying multivariate analyses methods to both surface EEG and iEEG recordings.

Schoenauer *et al*. [119] were the first who set out to test whether multivariate analyses methods would provide the means to capture memory processes during human sleep. Participants encoded pictures of houses or faces before going to sleep. To test whether the content of the prior-learned material was reactivated, the authors aimed for decoding the categorical features of the learned material from sleep EEG recordings. Indeed, the authors accomplished to derive solely from the sleep data whether participants had viewed faces or houses during previous learning. This finding speaks strongly for the notion that memories corresponding to these categories were in some way re-processed during sleep, leading to distinguishable distributions of the temporo-spatial EEG patterns. Hence, this work provided initial evidence for the capability of multivariate analyses techniques to detect memory reactivation during human sleep. Still, the applied methods and the experimental rationale hindered the ability to determine the fine-grained interaction, such as the temporal relationship between reactivation processes and sleep-related oscillations.

Cairney *et al*. [120] specifically addressed this open question by combining TMR, which should significantly narrow down the search-space for reactivation processes (see box 3), and a correlational decoding procedure (i.e. RSA). In this study, participants associated words with pictures comprising either scenes or objects before taking a nap. During ensuing NREM sleep, parts of the previously learned words were presented again as reminder cues, to experimentally trigger reactivation processes. Words which were previously associated with pictures induced stronger sleep spindle activity when compared with unrelated ‘new’ words, indicating that the sleeping brain responded preferentially to the reminders. More critically, during the rise of sleep spindles, the category of the memories linked to the auditory cues (i.e. objects or scenes) could be reliably decoded. In addition, the precision of the decoding forecasted the behavioural impact of TMR, suggesting the functional significance of the detected reactivation events. Hence, both studies outlined above [119,120] established that patterns of EEG activity during sleep can be successfully used to detect signatures of memory reprocessing.

Box 3. **Targeted Memory Reactivation.**

Studies in both humans and rodent models have demonstrated that sensory stimulation can modify sleep-related memory reactivation. ‘Targeted memory reactivation’ (TMR) studies follow the rationale that reminder cues, which were previously associated with the learning experience, are presented again during subsequent NREM sleep to specifically trigger reactivation processes. This approach has repeatedly proven successful, as both olfactory [121] and auditory cues (sounds, melodies, words [121–124]) have been shown to strengthen later memory performance for the cued memory representations [125]. Moreover, TMR has also turned out to represent a promising tool to elucidate the neural mechanisms of sleep-associated memory reactivation.

As an important proof of principle, work in rodents demonstrated that TMR cues indeed possess the capability to trigger memory reactivation, both in the hippocampus as well as in the neocortex [19,20]. Building upon these results, previous studies in humans mainly leveraged TMR to isolate the oscillatory scaffolding orchestrating memory reactivation during sleep. Accordingly, an intimate relationship between TMR and sleep spindles has been established. Promoting memory reactivation via TMR has been demonstrated to cause a robust surge in sleep spindle activity [120,126–130]. In addition, a growing number of TMR studies also point towards a critical role of theta with regards to memory reactivation during sleep. As with sleep spindles, cue-induced spectral theta power has been shown to be tightly linked to successful memory cueing of declarative memories [126,128–132], while theta also seems to modulate cue-related functional connectivity between brain regions implicated in the consolidation of motor memories [133]. Taken together, all of these studies established that TMR constitutes an effective tool to bias reactivation and allows accessing control over processes, which are typically hard to capture in humans.

A prevailing assumption touches on the similarity between learning and retrieval-related brain activation during wakefulness and ensuing reactivation patterns during sleep. If memory traces are composed of unique, distributed circuits of neuronal ensembles, (re)activation of a given representation during different states, be it the encoding or retrieval of memories during wakefulness or their reactivation during sleep, should be represented by highly similar, distributed neural activity [95]. Thus, a profound attempt to identify and characterize reactivation processes acting during sleep is ultimately bound to take learning and retrieval-related brain activity into account, which would also allow for a content-specific tracking of memory reactivation. As outlined above, early work in humans primarily tested whether EEG data recorded during sleep would comprise activity patterns that would permit discriminating stimulus categories that were present during encoding. This analytical rational represents without doubt a valid way to approximate memory processing during sleep. Still, the obtained results do not necessarily prove memory reactivation in its actual definition, as a direct test of whether learning/retrieval-related brain activity was reactivated during sleep was outside the scope of these studies.

To address this critical issue, Schreiner and colleagues [28] built upon previous findings indicating that the phase of low-frequency oscillations carries unique information about the content of sensory stimuli [134]. This feature of oscillatory phase has already been exploited to reveal reactivation processes during wakefulness, as it has been shown that encoding and retrieving the same memory content is signified by the emergence of similar phase patterns [29,46–48]. Hence, oscillatory phase might also constitute a prime candidate to uncover the reactivation of distinct memory traces during sleep and to assess their resemblance with reactivation processes tied to wakefulness.

In this study, participants performed a vocabulary-learning task in the evening, learning to associate Dutch words with their German translations [28]. Afterwards, memory performance for each association was assessed twice in two consecutive retrieval blocks. During ensuing NREM sleep, some of the prior learned Dutch words were presented again to the participants, following the rationale of previous TMR studies. In a first step, the authors tested whether retrieval of the same memory content during successive retrieval tests would be indexed by similar phase patterns, speaking not only for the content-specificity of low-frequency phase but also for its organizing role in retrieval-associated memory reactivation. Indeed, successfully remembering the same words when compared with non-remembered content was accompanied by elevated phase similarity in the theta range (5 Hz), suggesting that theta phase might coordinate the retrieval-induced reactivation of distinct memories. The most pressing question, however, was whether these content-specific features would also be traceable with regards to TMR-induced reactivation processes during sleep, indicating item-specific memory reactivation during sleep. To capture such TMR evoked memory reactivation, phase similarity for the same memory content was estimated again, but this time comparing the activity related to pre-sleep memory retrieval and TMR related activity during sleep. This analysis indeed yielded strong evidence that TMR-induced distinct reactivation processes, as retrieval-related phase patterns at 5 Hz, re-appeared in response to TMR reminders. Moreover, after the onset of TMR cues, these phase-coded reactivation patterns spontaneously fluctuated at a rate of 1 Hz, hinting towards a supra-ordinate coordination of the reactivation processes by SOs. In sum, those results demonstrated the close relatedness of cue-triggered memory reactivation during wakefulness and sleep, with theta oscillations orchestrating the re-processing of memories during both physiological states.

Moving beyond the declarative memory system, Belal *et al*. [135] set out to identify the sleep-related reactivation of procedural memories. Participants engaged in a motor memory task (SRTT [136]), which was followed by an imagery period where participants had to imagine the prior learned movements. During subsequent NREM sleep, tones associated with the learning of the task were presented again to elicit reactivation processes. The authors applied a multivariate classifier, which was trained on the imagery period, to the TMR data to detect reactivation of motor memories. In line with the findings on declarative memories [28,119,120], motor memory reactivation could be readily detected using multivariate analysis approaches, speaking for the general validity of these procedures.

While surface EEG recordings have provided precious insight into the memory function of the sleeping brain, a major limitation is its restricted spatial resolution. This constraint not only hinders the ability to assess the specific reactivation-related contributions of potential key areas such as the hippocampus and its subfields or the thalamus; it also prevents clarification of the association between hippocampal ripples and reactivation events in humans, which has been consistently observed in animal models. Thus, taking the impact of hippocampal ripples into consideration might significantly boost the information details that might be decoded from reactivated memory representations and thereby open a new window into investigating the memory function of the sleeping brain.

Recently, Zhang *et al*. [63] leveraged the opportunity of iEEG to probe the relationship of memory reactivation during wakefulness and sleep and the accompanying hippocampal ripples. Pre-surgical epilepsy patients learned pictures of houses or landscapes before they took a nap or stayed awake. After the 1 h sleep interval, they learned a new set of pictures and subsequently memory performance for all prior-learned pictures was assessed. To isolate stimulus-specific memory patterns, the authors assessed the degree of similarity in gamma activity (30–90 Hz) when the same memory content was learned and retrieved, when compared with inconsistent memory content. Here, higher similarity values were found for same memories, suggesting the presence of gamma-coded reactivation during retrieval.

To identify the spontaneous reactivation of memory representations, similarity between the stimulus-specific encoding patterns and brain activity accompanying quiet rest and sleep was evaluated. Intriguingly, encoding related brain activity was detectable to a comparable extent during both wakeful rest and sleep. Still, during both physiological states, the specified reactivation levels did not predict subsequent memory performance. Next, the authors set out to specifically assess the role of hippocampal ripples for the reactivation of memories. Reactivation processes during NREM sleep preferentially emerged during the presence of ripples, while no such association was observable during quiet rest. In addition, ripple-triggered reactivation particularly comprised later remembered memory content, indicating the necessity of this interplay for memory formation. In sum, this study elegantly revealed the critical role of hippocampal ripples for memory consolidation in humans. While memory reactivation was found to occur spontaneously (i) during both wakefulness and sleep and (ii) during and beyond the presence of hippocampal ripples, only ripple-triggered reactivation during NREM sleep impacted memory formation. These results clearly suggest that ripple-triggered memory reactivation might drive the memory function of sleep and further indicate the excellence of invasive recordings to study sleep-related memory processes.

## 4. Discussion and open questions

### (a) The when and where of memory reactivation

The evidence about the exact timing of memory reactivation within a retrieval process is equivocal. MEG/EEG and TMS studies characterized very early signatures of memory reactivation in the first 500 ms after cue onset [28,29,39,40,43,47,50]. Accordingly, some iEEG studies also found such early reactivation signatures [52], while others reported later onsets [55,69]. Importantly, the described reactivation signals were typically found in cortical sites, depending on the type of encoded content (e.g. sensory-specific reinstatement in sensory cortex, [29]). There is also recent evidence for a hippocampal-cortical dialogue during memory reactivation, in line with theoretical models and rodent work [2–4,8,137] suggesting that hippocampal pattern completion is followed by (cortical) reinstatement [55,69]. Assuming this sequence of processes, very early cortical reinstatement as described in several studies would call for an even earlier hippocampal pattern completion process, for which there is no supporting evidence so far [54,55,69]. Alternatively, the early cortical reactivation patterns could reflect the first stage of a cortico-hippocampal-cortical loop that has recently been suggested to underlie multi-sensory memory reactivation during sleep [20,138]. More insight on the temporal dynamics of memory reactivation processes comes from EEG work showing that reactivation not only occurs at a specific phase of a source-localized hippocampal 8 Hz rhythm, but is shifted 180° with respect to encoding related activity [42]. The results nicely confirm an oscillatory account of memory encoding-retrieval dynamics in the hippocampus [139].

Another open question is how high-frequency ripples time the reactivation of memory content. Rodent studies have shown reactivation during ripples in the context of spatial navigation [13,14,140,141], while studies investigating memory retrieval in humans demonstrated reactivation after ripple events [61,63], but not vice versa. Investigating this question would entail identifying how (e.g. compressed versus non-compressed) and where (hippocampus or neocortex) specific experiences (e.g. spatial versus non-spatial) are reactivated in the human brain. Investigating ripple-locked single-unit activity during human episodic memory retrieval could be a promising approach.

These studies could also elucidate whether replay during ripples and memory reinstatement after ripples serve different functions for remembering. Replay of spatial information during ripples in rodents is time-compressed [13,14,140], while retrieval-related memory reactivation after ripples in humans is not [61,63]. A recent human MEG study, however, provided first evidence for the coincidence of ripple-like activity and the time-compressed replay of sequences [86]. In contrast to the studies reporting non-compressed memory reactivation after ripples, this work investigated spontaneous replay of abstract sequences during rest. One could speculate that a non-compressed reactivation of memories after ripples provides the neural substrate necessary to consciously access these memories [57]. How non-compressed memory reactivation during sleep [28] fits into such an account remains an open question.

### (b) Is memory reactivation in humans compressed?

Should we expect to find compressed replay in humans, as has been shown in rodents [141]? While the majority of human reactivation studies did not directly address this question, there are reports of compressed memory reactivation in humans. Yaffe *et al*. [53] showed that the reactivation of distributed patterns of intracranially recorded high gamma activity at retrieval is compressed relative to its occurrence during encoding (see also [73]). Michelmann *et al*. [49] recorded MEG while participants remembered movie scenes, consisting of multiple sub-events, in order to track the speed and time-scale of memory reactivation. The authors reported evidence for a flexible switching between compressed and non-compressed forward reactivation of sequences during memory reactivation. Compressed replay has also been demonstrated during rest [86,87]. It remains an open question, however, whether compressed memory reactivation is suitable to create consciously accessible episodic memories [57]. Vividly remembering past events, the ‘mental travel to the past’, is at least not experienced as very compressed. It could still be the case that the actual reactivation of neural activity is compressed, but its interpretation (and with it, its experience) is not, and a rapid jump among snippets of episodes seems also plausible [49]. Alternatively, compressed and non-compressed reactivation could be functionally different, with the non-compressed version being read out only during episodic remembering and the compressed version used for planning and decision making.

### (c) When is memory replay in humans reversed?

Hippocampal sequences in rodents can be replayed forward and backward [140,142]. The particular direction of the replay has been suggested to vary depending on which cognitive process is using the replayed information [10,19]. In humans, backward replay has been demonstrated during rest [86,87] and working memory maintenance [81]. Interestingly, Liu *et al*. [86] recently demonstrated that the forward replay of learned sequences at rest can be reversed by providing a reward, indicating that the direction of replay might indeed be related to task goals [10,19]. In a recent EEG study, Linde-Domingo *et al*. [41] investigated the temporal structure during the reactivation of visual memories. The authors could show that the order during retrieval is reversed when compared with encoding: semantic features were reactivated before perceptual features. While this finding is not homologous to reversed memory replay in the rodent hippocampus, which usually does not involve different levels of hierarchies of representations, it shows that the order of a sequence can be reversed during memory readout. Since there are also reports of forward replay during memory retrieval [48], more research needs to be done to disentangle potentially different functions of forward and backward replay.

### (d) Comparing the electrophysiology of memory reactivation during sleep and wakefulness

#### (i) Oscillatory signatures of memory reactivation across states

Recent studies investigating the reactivation of memory content during memory retrieval converge on the view that the phase of low-frequency oscillatory activity can track item-specific memory reactivation. These studies are based on the assumption that the frequency and phase of oscillations represent information at a high level of specificity. Accordingly, content-specific oscillatory signatures should also be identifiable in the sleeping brain when memory is reactivated. The study by Schreiner *et al*. [28] provides the first promising results that this actually is the case. The same oscillatory signatures indexed memory reactivation during wakefulness and sleep, constituting first evidence for item-specific memory reactivation during human sleep. Future studies could exploit these commonalities to (i) uncover the fine-grained interplay of the reactivation signals with sleep-specific oscillations and to (ii) dissect potential functional differences with regards to memory reactivation during both physiological states.

#### (ii) The role of the thalamus during sleep and wakefulness

A prominent electrophysiological difference between wakefulness and sleep can be found in thalamocortical activity. During sleep, thalamic activity, which regulates sensory input to pass first-order thalamic nuclei and to arrive in the cortex, is governed by cortically generated SOs (less than 1 Hz) [143]. The gating function of the thalamus has been tightly linked to the emergence of SO-coupled sleep spindles [144], with burst firing of thalamocortical neurons during spindles blocking the processing of external sensations [144,145].

Indeed, it has been shown that the presence of sleep spindles specifically hinders the transmission of external stimuli from the thalamus to cortical areas [146].

Thus, by inducing cellular processes in favour of long-term potentiation and simultaneously deafferenting the cortex from external (sensory) inputs, sleep spindles might promote a neural environment, which enables undisturbed cortical reprocessing (i.e. reactivation) of hippocampal relayed memories [147]. In support of this notion, Halassa *et al*. [148] showed in mice that first-order thalamocortical neurons were inhibited during sleep spindles, reflecting thalamic gating of sensory input to the cortex. At the same time, higher-order, anterior thalamocortical neurons were not suppressed, potentially enabling offline processing of memories through their connections to the hippocampus and the frontal cortex. Thus, thalamic sleep spindles might be a key signature reflecting the balance between inhibition of sensory input and facilitation of mnemonic processes during sleep [149]. However, before specific thalamic and sleep spindle-related contributions to memory processes in humans might be discussed in full detail, several basic questions await further investigation. It is, for example, not known which and how thalamic nuclei participate in the generation of sleep spindles in humans and whether a comparable division of labour with regards to filtering external events and facilitating mnemonic processes exists.

The role of the human thalamus during memory processes while awake is not well understood. Evidence from human intracranial recordings point towards an involvement of the anterior and mediodorsal thalamus in memory processes [150–153], but a direct contribution of the human thalamus to memory reactivation still needs to be identified. Animal models describe an interaction between hippocampal ripple events and thalamic activity [154], but data from humans is lacking to date. A potential parallel between thalamic functions during wakefulness and sleep could be suppression of distraction. The frequency range of sleep spindles roughly corresponds to alpha/beta-band oscillations during wakefulness. Indeed it has been shown that sleep spindle-related neurons of the thalamic reticular nucleus are likewise associated to attention-related alpha oscillations during wakefulness, pointing towards a state independent thalamic inhibition principle [155].

Similar to the gating of information on a sensory level via alpha oscillations [156], prefrontal beta bursts might reflect an active inhibitory process to suppress distracting information at a higher level of the cortical hierarchy [157,158]. Intriguingly, beta oscillations have been suggested to reflect interactions between mediodorsal thalamus and prefrontal cortex during memory processes [150,159,160]. However, whether such thalamocortical beta activity plays an active role during memory reactivation while awake is an open question. Answers to these questions will depend on future studies that take the anatomical and functional intricacies of the thalamic micro-circuitry into account, which are, unfortunately, notoriously hard to dissect in humans.

#### (iii) Are high-frequency ripples a general marker for memory reactivation across brain states?

High-frequency ripples have been suggested to establish neuronal communication between the hippocampus and neocortex supporting the consolidation of memory traces [57–60,161]. In humans, hippocampal ripples (approx. 100 Hz) have been reported during sleep, quiet rest [63–65,162,163] and active waking periods [61]. Could such coupled ripple events index the coordination of memory reactivation (in the cortex) by the hippocampus, independent of the physiological state [12]?

While the available evidence from sleep studies seems to agree on a crucial role of high-frequency ripples for memory reactivation and consolidation [63–65], recent findings relating hippocampal ripples to awake memory retrieval seem to point in a similar direction.

Vaz *et al*. [61] provided evidence for ripple events that trigger memory reactivation in awake humans. Importantly, these ripple events were coupled between the medial temporal lobe and middle temporal gyrus and occurred before successful memory retrieval. Recent findings by Norman *et al*. [62] support the link between ripples and memory reactivation during free recall. Successfully remembered items were indexed by increased counts of hippocampal ripple events prior to their recall, which coincided with memory reactivation in visual areas. However, not all studies support this relationship. Zhang *et al*. [63] showed that only ripples recorded during non-REM sleep and not during the waking state triggered memory reactivation and predicted memory performance. It is important to note that the authors analysed spontaneous hippocampal ripples during rest, whereas Vaz *et al*. [61] and Norman *et al*. [62] analysed coupled ripples during active retrieval. One could, thus, speculate that there is a functional difference between coupled versus local ripples and between ripples during rest and active retrieval. However, ripples during awake memory retrieval seem to be rather rare events, and not every successful retrieval attempt is accompanied by coupled ripples [61]. More coupled ripples could be detected with direct recordings from the hippocampus or a larger spatial coverage (which is not easy to accomplish in human intracranial recordings), but for now the question remains whether high-frequency ripples consistently index memory reactivation during successful retrieval in humans. Recently, Liu *et al*. [86] identified ripple-like MEG activity localized to the medial temporal lobe which coincided with replay of previously learned abstract sequences. It would be highly interesting to see to what extent high-frequency MEG/EEG activity reflects ripples detected in intracranial data [61]. A non-invasive approximation of hippocampal ripples could be a very promising tool that would certainly inspire many researchers interested in memory processes during sleep and wakefulness.

#### (iv) Is awake memory reactivation sufficient for memory consolidation?

Several researchers have argued that reactivating a memory trace could either facilitate or induce memory consolidation [12,164]. Following this account, every successful retrieval from episodic memory should facilitate/induce memory consolidation. The testing effect shows that memory retrieval, when compared with re-learning, results in better memory performance [165,166]. This effect is particularly strong with longer retention intervals (e.g. greater than 24 h), leaving the question whether sleep is exclusively needed for consolidation unanswered. The first evidence for a ‘fast route to memory consolidation’ irrespective of sleep comes from a recent fMRI study reporting a rapid emergence of memory engrams in the parietal cortex [167], but human electrophysiological evidence is still lacking.

## Data accessibility

This article has no additional data.
